# 2,4,6-Trinitro­phenyl 3-methyl­benzoate

**DOI:** 10.1107/S1600536812027407

**Published:** 2012-06-23

**Authors:** Rodolfo Moreno-Fuquen, Fabricio Mosquera, Javier Ellena, Juan C. Tenorio

**Affiliations:** a Departamento de Química – Facultad de Ciencias, Universidad del Valle, Apartado 25360, Santiago de Cali, Colombia; b Instituto de Física de São Carlos, IFSC, Universidade de São Paulo, USP, São Carlos, SP, Brazil

## Abstract

In the title benzoate derivative, C_14_H_9_N_3_O_8_, the benzene rings form a dihedral angle of 87.48 (5)°. The central ester unit forms an angle of 19.42 (7)° with the methyl­benzene ring, indicating a significant twist. In the crystal, the mol­ecules are linked by weak C—H⋯O inter­actions forming a helical chain along [010].

## Related literature

For synthesis of picric acid with charge-transfer complexes, see: Siddaraju *et al.* (2012 ▶); Refat *et al.* (2010 ▶); El-Medania *et al.* (2003 ▶). For the pharmacological and biochemical activity of picric acid, see: Khan & Ovesb (2010 ▶); Khan *et al.* (2011 ▶). For the non-linear optical properties of picric acid, see: Zaderenko *et al.* (1997 ▶). For the synthesis of nitro­aromatic compounds with industrial use, see: Ju & Parales (2010 ▶). For similar structures, see: Adams & Morsi (1976 ▶); Gowda *et al.* (2007 ▶, 2008 ▶, 2009 ▶). For hydrogen bonding, see: Nardelli (1995 ▶). For hydrogen-bond graph-set motifs, see: Etter (1990 ▶).
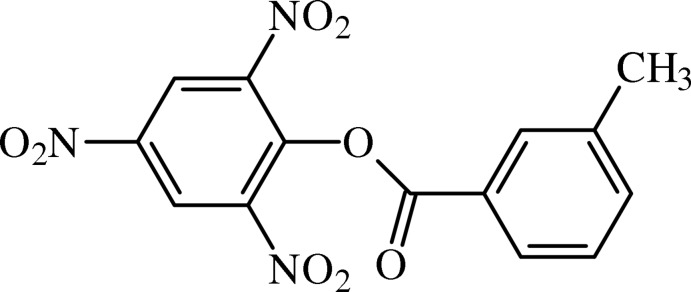


## Experimental

### 

#### Crystal data

C_14_H_9_N_3_O_8_
*M* *_r_* = 347.24
Monoclinic, 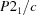

*a* = 7.4947 (1) Å
*b* = 8.4366 (2) Å
*c* = 23.8574 (6) Å
β = 99.365 (1)°
*V* = 1488.39 (6) Å^3^
*Z* = 4
Mo *K*α radiation
μ = 0.13 mm^−1^
*T* = 295 K
0.38 × 0.34 × 0.28 mm

#### Data collection

Nonius KappaCCD diffractometer
5874 measured reflections
3043 independent reflections
2373 reflections with *I* > 2σ(*I*)
*R*_int_ = 0.016

#### Refinement

*R*[*F* ^2^ > 2σ(*F* ^2^)] = 0.044
*wR*(*F* ^2^) = 0.126
*S* = 1.04
3043 reflections
226 parameters
1 restraint
H-atom parameters constrained
Δρ_max_ = 0.19 e Å^−3^
Δρ_min_ = −0.22 e Å^−3^

### 

Data collection: *COLLECT* (Nonius, 2000 ▶); cell refinement: *SCALEPACK* (Otwinowski & Minor, 1997 ▶); data reduction: *DENZO* (Otwinowski & Minor, 1997 ▶) and *SCALEPACK*; program(s) used to solve structure: *SHELXS97* (Sheldrick, 2008 ▶); program(s) used to refine structure: *SHELXL97* (Sheldrick, 2008 ▶); molecular graphics: *ORTEP-3 for Windows* (Farrugia, 1997 ▶) and *Mercury* (Macrae *et al.*, 2006 ▶); software used to prepare material for publication: *WinGX* (Farrugia, 1999 ▶).

## Supplementary Material

Crystal structure: contains datablock(s) I, global. DOI: 10.1107/S1600536812027407/tk5112sup1.cif

Structure factors: contains datablock(s) I. DOI: 10.1107/S1600536812027407/tk5112Isup2.hkl

Supplementary material file. DOI: 10.1107/S1600536812027407/tk5112Isup3.cml

Additional supplementary materials:  crystallographic information; 3D view; checkCIF report

## Figures and Tables

**Table 1:** Hydrogen-bond geometry (Å, °)

*D*—H⋯*A*	*D*—H	H⋯*A*	*D*⋯*A*	*D*—H⋯*A*
C3—H3⋯O8^i^	0.93	2.50	3.4276 (19)	176
C13—H13⋯O3^ii^	0.93	2.70	3.346 (2)	127

Symmetry codes: (i) 
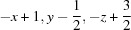
; (ii) 
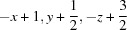
.

**Fig. 1.**
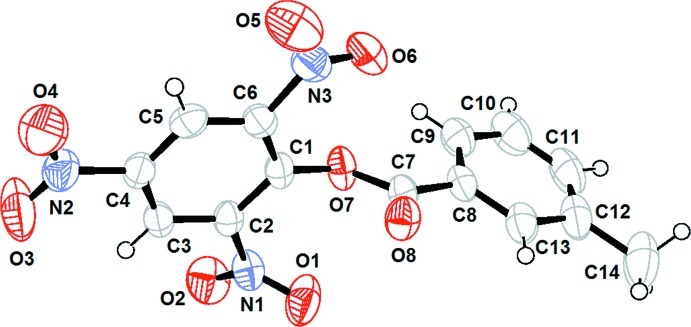
Molecular conformation and atom numbering scheme for the title compound with displacement ellipsoids drawn at the 50% probability level. H atoms are shown as spheres of arbitrary radius.

**Fig. 2.**
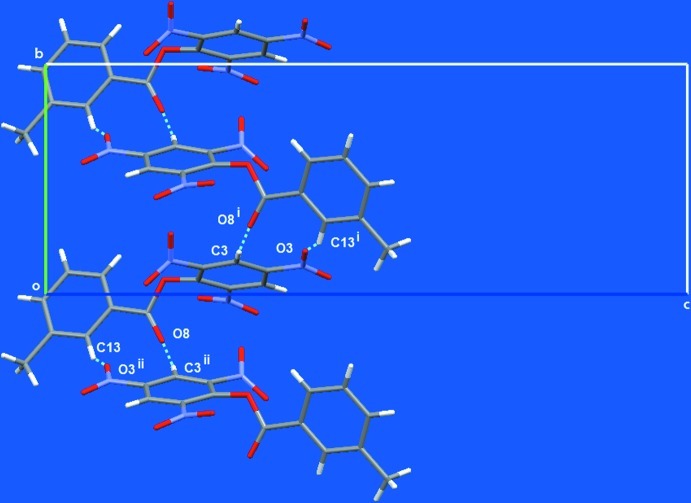
Part of the crystal structure of (I), showing the formation of chains running along [010]. Symmetry code: (i) -x - 1,+y - 1/2,-z - 1/2; (ii) -x - 1,+y + 1/2,-z - 1/2.

## supplementary crystallographic information

### Comment

The structure determination of 2,4,6-trinitrophenyl 3-methylbenzoate, (I), is part of a series of studies on novel nitro aryl benzoates, carried out in our research group. This molecular system is strongly defined by the presence of the phenyl moiety coming from picric acid (TNP), which has not been employed previously in the synthesis of the title ester. TNP is interesting because of its widespread use in the synthesis of charge transfer complexes, which often tend to have significant crystalline properties (Siddaraju *et al.*, 2012; Refat *et al.*, 2010; El-Medania *et al.*, 2003), pharmacological activity as anti-microbial agents and DNA-binding systems (Khan & Ovesb, 2010; Khan *et al.*, 2011) and good non-linear optical (NLO) properties (Zaderenko *et al.*, 1997). However, the properties of esters having the TNP moiety remain largely unknown. Therefore, this research will present a new compound, (I), with the aim to understand its properties. Moreover, is well known that nitroaromatics and their derivatives constitute a main class of industrial chemicals and are widely used as intermediates in the synthesis of many varied products, ranging from drugs, pigments, pesticides and plant growth regulators to the explosives (Ju & Parales, 2010).

The molecular structure of (I) is shown in Fig. 1. Bond lengths and bond angles of (I) show marked similarity with other aryl benzoates reported in the literature such as phenyl benzoate (PBA) (Adams & Morsi, 1976), 3-methylphenyl benzoate (3MePBA) (Gowda *et al.*, 2007), 2,4-dimethylphenyl benzoate (24DMPBA) (Gowda *et al.*, 2008), 2.5-dimethylphenyl benzoate (25DMPBA) (Gowda *et al.*, 2009), among others. The benzene rings of (I) form a dihedral angle of 87.48 (5)°, a value which is quite consistent with other aryl benzoate systems such as 25DMPBA, 3MePBA and 24DMPBA which present dihedral angles of 87.4 (1), 80.3 (1) and 79.61 (6)°, respectively. The central ester moiety forms an angle of 19.42 (7)° with the methylbenzene ring to which it is attached. The nitro groups form dihedral angles with the benzene ring to which they are attached of 43.15 (10), 7.72 (14) and 13.56 (18)° for O1—N1—O2, O3—N2—O4 and O5—N3—O6, respectively.

The molecules are packed through weak C—H···O interactions forming one-dimensional helical chains along [010] (see Table 1, Nardelli, 1995). These intermolecular contacts are explained in terms of the substructure shown in Fig. 2. The C3 atom of the phenyl ring at (*x*,*y*,*z*) acts as a hydrogen-bond donor to carbonyl atom O8 at (-*x*-1, +*y*-1/2, -*z*-1/2). Growth in this direction is reinforced by the weak C13—H13···O3 interaction, in which the C13 atom of the benzoate ring at (*x*,*y*,*z*) acts as hydrogen-bond donor to atom O3 from one of the nitro groups at (-*x*-1, +*y*+1/2, -*z*-1/2). The combination of these two contacts generate edge-fused rings *R*^2^_2_(10) (Etter, 1990), along [010].

### Experimental

The reagents and solvents for the synthesis were obtained from the Aldrich Chemical Co., and were used without additional purification. The title molecule was obtained through a two-step reaction. First, 3-methylbenzoic acid (0.25 g, 1.838 mmol) was refluxed in excess thionyl chloride (10 ml) for an hour. Then, the thionyl chloride was distilled off under reduced pressure to purify the 3-methylbenzoyl chloride obtained as a pale-yellow translucent liquid. The same reaction flask was rearranged and a solution of picric acid (0.42 g, 1.835 mmol) in acetonitrile was dropped inside it with constant stirring. The reaction mixture was taken to reflux for about an hour. A pale-yellow solid was obtained after leaving the solvent to evaporate. This was washed with distilled water and cold methanol to eliminate impurities. Crystals of good quality and suitable for single-crystal X-ray diffraction were grown in acetonitrile. IR spectra was recorded on a FT—IR SHIMADZU IR-Affinity-1 spectrophotometer. Pale-yellow crystals; yield 73%; *M*.pt: 425 (1) K. IR (KBr, cm^-1^) 3114.37, 3086.94 (aromatic C—H); 2957.26, 2920.94 (methyl C—H); 1752.24 (ester C═O); 1613.04 (C═); 1547.90, 1343.62 (–NO_2_); 1234.22 (C(═O)—O).

### Refinement

All H-atoms were positioned at geometrically idealized positions with C—H distance of 0.93 Å and *U*_iso_(H) = 1.2 times *U*_eq_ of the C-atoms to which they were bonded.

### Crystal data

**Table:**

C_14_H_9_N_3_O_8_	*F*(000) = 712
*M**_r_* = 347.24	*D*_x_ = 1.550 Mg m^−^^3^
Monoclinic, *P*2_1_/*c*	Melting point: 425(1) K
Hall symbol: -P 2ybc	Mo *K*α radiation, λ = 0.71073 Å
*a* = 7.4947 (1) Å	Cell parameters from 5756 reflections
*b* = 8.4366 (2) Å	θ = 3.0–26.4°
*c* = 23.8574 (6) Å	µ = 0.13 mm^−^^1^
β = 99.365 (1)°	*T* = 295 K
*V* = 1488.39 (6) Å^3^	Block, pale-yellow
*Z* = 4	0.38 × 0.34 × 0.28 mm

### Data collection

**Table:**

Nonius KappaCCD diffractometer	2373 reflections with *I* > 2σ(*I*)
Radiation source: fine-focus sealed tube	*R*_int_ = 0.016
Graphite monochromator	θ_max_ = 26.4°, θ_min_ = 3.0°
CCD rotation images, thick slices scans	*h* = −9→9
5874 measured reflections	*k* = −10→10
3043 independent reflections	*l* = −29→29

### Refinement

**Table:**

Refinement on *F*^2^	Primary atom site location: structure-invariant direct methods
Least-squares matrix: full	Secondary atom site location: difference Fourier map
*R*[*F*^2^ > 2σ(*F*^2^)] = 0.044	Hydrogen site location: inferred from neighbouring sites
*wR*(*F*^2^) = 0.126	H-atom parameters constrained
*S* = 1.04	*w* = 1/[σ^2^(*F*_o_^2^) + (0.0676*P*)^2^ + 0.2831*P*] where *P* = (*F*_o_^2^ + 2*F*_c_^2^)/3
3043 reflections	(Δ/σ)_max_ < 0.001
226 parameters	Δρ_max_ = 0.19 e Å^−^^3^
1 restraint	Δρ_min_ = −0.22 e Å^−^^3^

### Special details

**Table:**

Geometry. All esds (except the esd in the dihedral angle between two l.s. planes) are estimated using the full covariance matrix. The cell esds are taken into account individually in the estimation of esds in distances, angles and torsion angles; correlations between esds in cell parameters are only used when they are defined by crystal symmetry. An approximate (isotropic) treatment of cell esds is used for estimating esds involving l.s. planes.
Refinement. Refinement of *F*^2^ against ALL reflections. The weighted *R*-factor *wR* and goodness of fit *S* are based on *F*^2^, conventional *R*-factors *R* are based on *F*, with *F* set to zero for negative *F*^2^. The threshold expression of *F*^2^ > σ(*F*^2^) is used only for calculating *R*-factors(gt) *etc*. and is not relevant to the choice of reflections for refinement. *R*-factors based on *F*^2^ are statistically about twice as large as those based on *F*, and *R*- factors based on ALL data will be even larger.

### Fractional atomic coordinates and isotropic or equivalent isotropic displacement parameters (Å^2^)

**Table:**

	*x*	*y*	*z*	*U*_iso_*/*U*_eq_	
O7	0.90799 (14)	0.93604 (12)	0.81583 (5)	0.0483 (3)	
O8	0.89917 (15)	1.20129 (13)	0.81861 (5)	0.0505 (3)	
C1	0.80954 (19)	0.93669 (16)	0.76227 (6)	0.0391 (3)	
C5	0.7782 (2)	0.95541 (18)	0.66027 (7)	0.0454 (4)	
H5	0.8253	0.9833	0.6279	0.054*	
C3	0.5283 (2)	0.85846 (18)	0.70267 (6)	0.0420 (4)	
H3	0.4117	0.8176	0.6989	0.050*	
C7	0.95268 (19)	1.08060 (17)	0.84158 (6)	0.0394 (3)	
C2	0.63119 (19)	0.88363 (17)	0.75520 (6)	0.0392 (3)	
O6	1.17307 (17)	1.0163 (2)	0.75989 (6)	0.0837 (5)	
C4	0.6059 (2)	0.89658 (18)	0.65603 (6)	0.0440 (4)	
N1	0.54579 (18)	0.85492 (17)	0.80541 (6)	0.0503 (4)	
C8	1.0651 (2)	1.05927 (19)	0.89762 (6)	0.0440 (4)	
C6	0.88038 (19)	0.97246 (17)	0.71349 (7)	0.0423 (4)	
O2	0.45102 (19)	0.73845 (17)	0.80489 (6)	0.0738 (4)	
N3	1.06867 (19)	1.02766 (17)	0.71553 (7)	0.0549 (4)	
N2	0.4983 (2)	0.8763 (2)	0.59912 (6)	0.0605 (4)	
O1	0.5703 (2)	0.95298 (19)	0.84340 (5)	0.0800 (5)	
C13	1.0733 (2)	1.1822 (2)	0.93617 (7)	0.0534 (4)	
H13	1.0100	1.2754	0.9260	0.064*	
O4	0.5610 (2)	0.9263 (2)	0.55895 (6)	0.0926 (5)	
C9	1.1597 (2)	0.9197 (2)	0.91180 (8)	0.0561 (4)	
H9	1.1550	0.8373	0.8857	0.067*	
O5	1.1103 (2)	1.07925 (18)	0.67223 (7)	0.0818 (5)	
O3	0.3529 (2)	0.8124 (2)	0.59560 (6)	0.0989 (6)	
C10	1.2612 (3)	0.9054 (3)	0.96550 (9)	0.0725 (6)	
H10	1.3260	0.8129	0.9756	0.087*	
C12	1.1757 (3)	1.1678 (3)	0.99043 (7)	0.0637 (5)	
C11	1.2666 (3)	1.0267 (3)	1.00373 (8)	0.0739 (6)	
H11	1.3335	1.0139	1.0398	0.089*	
C14	1.1835 (4)	1.3021 (3)	1.03270 (9)	0.0947 (8)	
H14A	1.1121	1.3893	1.0156	0.142*	
H14B	1.3066	1.3358	1.0436	0.142*	
H14C	1.1367	1.2667	1.0657	0.142*	

### Atomic displacement parameters (Å^2^)

**Table:**

	*U*^11^	*U*^22^	*U*^33^	*U*^12^	*U*^13^	*U*^23^
O7	0.0535 (6)	0.0349 (6)	0.0487 (6)	−0.0045 (4)	−0.0147 (5)	0.0028 (5)
O8	0.0610 (7)	0.0369 (6)	0.0502 (6)	−0.0019 (5)	−0.0013 (5)	0.0013 (5)
C1	0.0426 (8)	0.0275 (7)	0.0432 (8)	0.0017 (6)	−0.0056 (6)	−0.0010 (6)
C5	0.0509 (9)	0.0403 (8)	0.0461 (9)	0.0085 (7)	0.0115 (7)	−0.0015 (7)
C3	0.0398 (7)	0.0385 (8)	0.0444 (8)	−0.0002 (6)	−0.0028 (6)	−0.0039 (6)
C7	0.0369 (7)	0.0375 (8)	0.0425 (8)	−0.0071 (6)	0.0030 (6)	0.0001 (6)
C2	0.0439 (8)	0.0318 (7)	0.0397 (8)	−0.0014 (6)	0.0002 (6)	0.0002 (6)
O6	0.0411 (7)	0.1268 (14)	0.0802 (10)	−0.0085 (7)	0.0011 (7)	−0.0216 (9)
C4	0.0473 (8)	0.0421 (8)	0.0395 (8)	0.0081 (6)	−0.0020 (6)	−0.0064 (6)
N1	0.0515 (8)	0.0516 (8)	0.0451 (8)	−0.0095 (7)	−0.0002 (6)	0.0054 (6)
C8	0.0380 (7)	0.0521 (9)	0.0402 (8)	−0.0120 (7)	0.0011 (6)	0.0028 (7)
C6	0.0386 (7)	0.0328 (7)	0.0543 (9)	0.0026 (6)	0.0040 (7)	−0.0036 (6)
O2	0.0792 (9)	0.0595 (8)	0.0852 (10)	−0.0265 (7)	0.0204 (7)	0.0087 (7)
N3	0.0453 (8)	0.0413 (8)	0.0786 (11)	0.0013 (6)	0.0114 (8)	−0.0086 (7)
N2	0.0637 (10)	0.0724 (10)	0.0417 (8)	0.0131 (8)	−0.0023 (7)	−0.0065 (7)
O1	0.0935 (10)	0.1009 (12)	0.0478 (7)	−0.0366 (9)	0.0178 (7)	−0.0204 (8)
C13	0.0520 (9)	0.0604 (10)	0.0471 (9)	−0.0178 (8)	0.0060 (7)	−0.0026 (8)
O4	0.0837 (10)	0.1518 (16)	0.0412 (7)	0.0160 (10)	0.0066 (7)	0.0088 (9)
C9	0.0489 (9)	0.0654 (11)	0.0502 (9)	−0.0012 (8)	−0.0035 (7)	0.0051 (8)
O5	0.0700 (9)	0.0748 (10)	0.1047 (12)	−0.0148 (7)	0.0267 (8)	0.0253 (8)
O3	0.0843 (11)	0.1433 (16)	0.0592 (9)	−0.0384 (11)	−0.0183 (7)	−0.0077 (9)
C10	0.0568 (11)	0.0928 (16)	0.0612 (12)	0.0017 (10)	−0.0103 (9)	0.0176 (11)
C12	0.0622 (11)	0.0871 (14)	0.0417 (9)	−0.0342 (11)	0.0078 (8)	−0.0066 (9)
C11	0.0560 (11)	0.1151 (18)	0.0453 (10)	−0.0233 (12)	−0.0081 (8)	0.0099 (11)
C14	0.1136 (18)	0.118 (2)	0.0529 (12)	−0.0513 (16)	0.0130 (11)	−0.0233 (12)

### Geometric parameters (Å, º)

**Table:**

O7—C1	1.3676 (17)	C8—C9	1.388 (2)
O7—C7	1.3820 (18)	C6—N3	1.479 (2)
O8—C7	1.1942 (18)	N3—O5	1.208 (2)
C1—C6	1.389 (2)	N2—O3	1.207 (2)
C1—C2	1.394 (2)	N2—O4	1.210 (2)
C5—C4	1.372 (2)	C13—C12	1.399 (2)
C5—C6	1.379 (2)	C13—H13	0.9300
C5—H5	0.9300	C9—C10	1.385 (2)
C3—C4	1.375 (2)	C9—H9	0.9300
C3—C2	1.377 (2)	C10—C11	1.367 (3)
C3—H3	0.9300	C10—H10	0.9300
C7—C8	1.471 (2)	C12—C11	1.382 (3)
C2—N1	1.467 (2)	C12—C14	1.512 (3)
O6—N3	1.2132 (19)	C11—H11	0.9300
C4—N2	1.472 (2)	C14—H14A	0.9600
N1—O2	1.2113 (18)	C14—H14B	0.9600
N1—O1	1.2188 (19)	C14—H14C	0.9600
C8—C13	1.381 (2)		
			
C1—O7—C7	117.82 (11)	O5—N3—O6	123.69 (16)
O7—C1—C6	124.20 (13)	O5—N3—C6	117.60 (15)
O7—C1—C2	118.21 (14)	O6—N3—C6	118.70 (15)
C6—C1—C2	117.29 (13)	O3—N2—O4	124.29 (16)
C4—C5—C6	118.72 (15)	O3—N2—C4	117.98 (16)
C4—C5—H5	120.6	O4—N2—C4	117.73 (16)
C6—C5—H5	120.6	C8—C13—C12	120.63 (18)
C4—C3—C2	116.89 (14)	C8—C13—H13	119.7
C4—C3—H3	121.6	C12—C13—H13	119.7
C2—C3—H3	121.6	C10—C9—C8	118.80 (18)
O8—C7—O7	120.62 (13)	C10—C9—H9	120.6
O8—C7—C8	128.42 (14)	C8—C9—H9	120.6
O7—C7—C8	110.95 (12)	C11—C10—C9	120.3 (2)
C3—C2—C1	122.95 (14)	C11—C10—H10	119.9
C3—C2—N1	117.60 (13)	C9—C10—H10	119.9
C1—C2—N1	119.44 (13)	C11—C12—C13	117.58 (18)
C5—C4—C3	122.82 (14)	C11—C12—C14	121.89 (19)
C5—C4—N2	118.55 (15)	C13—C12—C14	120.5 (2)
C3—C4—N2	118.61 (14)	C10—C11—C12	122.12 (17)
O2—N1—O1	125.12 (15)	C10—C11—H11	118.9
O2—N1—C2	117.30 (14)	C12—C11—H11	118.9
O1—N1—C2	117.52 (13)	C12—C14—H14A	109.5
C13—C8—C9	120.61 (15)	C12—C14—H14B	109.5
C13—C8—C7	118.07 (15)	H14A—C14—H14B	109.5
C9—C8—C7	121.32 (15)	C12—C14—H14C	109.5
C5—C6—C1	121.20 (14)	H14A—C14—H14C	109.5
C5—C6—N3	116.54 (15)	H14B—C14—H14C	109.5
C1—C6—N3	122.25 (14)		
			
C7—O7—C1—C6	75.78 (19)	C4—C5—C6—N3	176.60 (13)
C7—O7—C1—C2	−110.65 (15)	O7—C1—C6—C5	173.36 (13)
C1—O7—C7—O8	3.5 (2)	C2—C1—C6—C5	−0.3 (2)
C1—O7—C7—C8	−177.40 (13)	O7—C1—C6—N3	−5.5 (2)
C4—C3—C2—C1	−3.5 (2)	C2—C1—C6—N3	−179.14 (13)
C4—C3—C2—N1	175.52 (13)	C5—C6—N3—O5	12.3 (2)
O7—C1—C2—C3	−170.71 (13)	C1—C6—N3—O5	−168.75 (15)
C6—C1—C2—C3	3.3 (2)	C5—C6—N3—O6	−166.39 (15)
O7—C1—C2—N1	10.3 (2)	C1—C6—N3—O6	12.5 (2)
C6—C1—C2—N1	−175.72 (13)	C5—C4—N2—O3	173.96 (18)
C6—C5—C4—C3	2.1 (2)	C3—C4—N2—O3	−7.2 (2)
C6—C5—C4—N2	−179.10 (13)	C5—C4—N2—O4	−6.7 (2)
C2—C3—C4—C5	0.7 (2)	C3—C4—N2—O4	172.15 (16)
C2—C3—C4—N2	−178.05 (13)	C9—C8—C13—C12	−0.6 (2)
C3—C2—N1—O2	41.7 (2)	C7—C8—C13—C12	179.01 (15)
C1—C2—N1—O2	−139.18 (15)	C13—C8—C9—C10	0.5 (3)
C3—C2—N1—O1	−135.63 (16)	C7—C8—C9—C10	−179.05 (16)
C1—C2—N1—O1	43.4 (2)	C8—C9—C10—C11	0.4 (3)
O8—C7—C8—C13	19.5 (2)	C8—C13—C12—C11	−0.3 (3)
O7—C7—C8—C13	−159.49 (14)	C8—C13—C12—C14	−179.62 (17)
O8—C7—C8—C9	−160.88 (17)	C9—C10—C11—C12	−1.3 (3)
O7—C7—C8—C9	20.1 (2)	C13—C12—C11—C10	1.3 (3)
C4—C5—C6—C1	−2.3 (2)	C14—C12—C11—C10	−179.43 (19)

### Hydrogen-bond geometry (Å, º)

**Table:**

*D*—H···*A*	*D*—H	H···*A*	*D*···*A*	*D*—H···*A*
C3—H3···O8^i^	0.93	2.50	3.4276 (19)	176
C13—H13···O3^ii^	0.93	2.70	3.346 (2)	127

Symmetry codes: (i) −*x*+1, *y*−1/2, −*z*+3/2; (ii) −*x*+1, *y*+1/2, −*z*+3/2.
